# Les tumeurs à cellules géantes des gaines synoviales de la main: à propos de 50 cas

**DOI:** 10.11604/pamj.2017.26.128.9514

**Published:** 2017-03-07

**Authors:** Walid Osman, Zeineb Alaya, Ali Haggui, Mohamed Ben Rejeb, Sonia Jemni, Nader Naouar, Mohamed Laziz Ben Ayeche

**Affiliations:** 1 Service de Chirurgie Orthopédique. Hôpital Sahloul, Sousse, Tunisie; 2 Service de Rhumatologie, Hôpital Farhat Hached, Sousse, Tunisie; 3 Service de Chirurgie Générale, Hôpital Régional de Kasserine, Tunisie; 4 Service d’Hygiène Hospitalière, Hôpital Sahloul, Sousse, Tunisie; 5 Service de Médecine Physique et Rééducation Fonctionnelle, Hôpital Sahloul, Sousse, Tunisie

**Keywords:** Gaine synoviale, tendon, tumeur à cellules géantes, main, chirurgie, Synovial sheath, tendon, giant cell tumor, hand, surgery

## Abstract

Les tumeurs à cellules géantes des gaines synoviales (TCGGS) des tendons représentent la forme localisée de la synovite villonodulaire hémopigmentée. Elles s'observent le plus souvent au niveau des mains. Notre but était d'étudier les caractéristiques épidémio-cliniques et thérapeutiques des TCGGS, évaluer les résultats du traitement chirurgical et dégager les facteurs de récidives. Il s'agit d'une étude rétrospective de 50 cas de TCGGS de la main colligés entre 1992 et 2016 au service d'orthopédie de l'hôpital Sahloul de Sousse en Tunisie. Les caractéristiques cliniques et épidémiologiques des TCGGS ont été précisées. L'âge moyen des patients était de 33 ans (9-69 ans) avec un sexe-ratio de 0,6. Les motifs de consultation étaient l'apparition constante d'une tuméfaction (100%), la gêne à la mobilisation des articulations inter-phalangiennes (6%) et la douleur digitale (18%). Toutes les tumeurs étaient localisées au niveau de la région digitale, surtout au niveau de l'index (42%). La localisation était palmaire dans 66% des cas. Tous les malades ont été opérés, l'aspect macroscopique montrait une tumeur encapsulée, polylobée et jaune brunâtre qui se prolongeait dans la gaine des tendons fléchisseurs (4 cas) et sous le tendon extenseur (2 cas). Nous avons noté un seul cas de récidive, soit 2% qui ont été repris chirurgicalement. Les résultats fonctionnels étaient bons dans tous les cas. Le diagnostic de TCGGS doit être évoqué devant une tuméfaction digitale. Leur prise en charge fait appel à la chirurgie qui reste difficile et doit être correctement exécutée pour éviter les récidives.

## Abstract (en)

Giant cell tumors of the synovial tendon sheaths (GCTSTS) are a localized form of hemopigmented villonodular synovitis. They mainly affect the hands. This study aims to analyse the epidemiology, clinical and therapeutic characteristics of GCTSTS, to assess the results of surgical treatment and to identify risk factors for recurrences. We conducted a retrospective data collection from medical records of 50 patients with GCTSTS of the hand between 1992 and 2016 in the Department of Orthopaedics at the Hospital of Sahloul (Sousse-Tunisia). The clinical and epidemiological features of GCTSTS have been specified. The average age of patients was 33 years (9-69 years), the sex ratio was 0,6. Constant swelling (100%), joints inter-phalangeal mobilization trouble (6%) and digital pain (18%) were the most frequent reason for consultation. All tumors were located at the level of the digital region, especially at the level of the index (42%). It occurred on the palm of the hand in 66% of cases. All patients underwent surgery; macroscopically GCTTS appeared as an encapsulated polylobed and yellowish brown tumor, extending into the flexor tendons sheath(4 cases) and under the extensor tendon (2 cases). We noted a single case of recurrent cancer (2%) which was treated surgically. The functional results were good in all cases. The diagnosis of GCTTS should be evoked when there is evidence of digital swelling. Their management is based on surgery which is difficult and should be performed correctly to avoid recurrences.

## Introduction

Les tumeurs à cellules géantes des gaines synoviales des tendons (TCGGS), ou tumeur ténosynoviale à cellules géantes, ou synovite villonodulaire hémopigmentée, se développent à partir de la synoviale articulaire et péri-articulaire. Ces tumeurs constituent un désordre prolifératif bénin de la synoviale dont l'étiopathogénie reste encore indéterminée. La théorie la plus largement admise est celle proposée par Jaffé et al [1], qui suggère une hyperplasie réactive ou régénératrice de la synoviale secondaire à un processus inflammatoire. C'est une tumeur bénigne fréquente au niveau de la main, deuxième par ordre de fréquence après le kyste synovial [2]. La TCGGS se présente comme une masse généralement unique, indolore à croissance lente qui peut s'étaler sur plusieurs années. L'évolution non douloureuse et peu invalidante explique la fréquence de tumeurs de taille importante lors de la première consultation, s'étendant parfois circonférenciellement. En dépit de son caractère bénin, les récidives sont fréquentes pouvant aller jusqu'à 45% [3] et la dégénérescence sous forme maligne n'a pas été rapportée dans la littérature. Plusieurs facteurs de récidive sont évoqués dans la littérature : la localisation au niveau de l'interphalangienne distale (IPD), la présence d´arthrose, les érosions de pression sur les radiographies, l'activité mitotique élevée et les lésions de type 2 décrites par Al-Qattan [2–5]. Le traitement qui repose sur une exérèse chirurgicale complète, est rendu difficile par l'envahissement diffus aux structures nobles de voisinage. Notre travail avait pour objectif d'étudier les caractéristiques épidémio-cliniques et thérapeutiques des TCGGS, évaluer les résultats du traitement chirurgical et dégager les facteurs de récidives.

## Méthodes

Nous rapportons 50 cas de TCGGS de la main, traitées et suivies au service d'orthopédie du CHU Sahloul de Sousse en Tunisie sur une période de vingt quatre ans (1992-2016). Nous avons établi une fiche d'exploitation pour tous les malades permettant d'étudier l'âge, le sexe, la circonstance de découverte, la durée d'évolution, les caractéristiques cliniques, radiologiques de la tumeur, le compte rendu opératoire, le résultat anatomopathologique, les éventuelles complications, la fonction du doigt opéré et enfin la survenue ou non d'une récidive. Les données épidémiologiques, cliniques, radiologiques et anatomo-pathologiques ont été récupérées des dossiers médicaux. Tous les patients ont été opérés sous anesthésie générale (AG) ou sous anesthésie plexique avec hémostase préventive par garrot pneumatique à la racine du membre supérieur. L'incision est palmaire en zigzag (de Brunner) permettant un repérage des pédicules collatéraux et du système fléchisseur du doigt. Cette voie offre une meilleure exposition de la tumeur. Après exérèse tumorale, la pièce opératoire est adressée pour un examen anatomo-pathologique. La fermeture est assurée par des points séparés après hémostase. L'immobilisation de la main est faite par une attelle en manchette en position intrinsèque pendant 2 à 3 semaines, date à laquelle l'ablation des points est réalisée. La rééducation est prescrite à cette date. Au dernier recul, le résultat du traitement chirurgical est évalué par la recherche d'une éventuelle récidive ou une autre localisation, par l'étude de la mobilité digitale, par la détection d'une éventuelle complication, et par l'appréciation de la qualité de la cicatrice. Microsoft Excel a été utilisé pour analyser les données statistiques simples.

## Résultats

**Figure 1:**
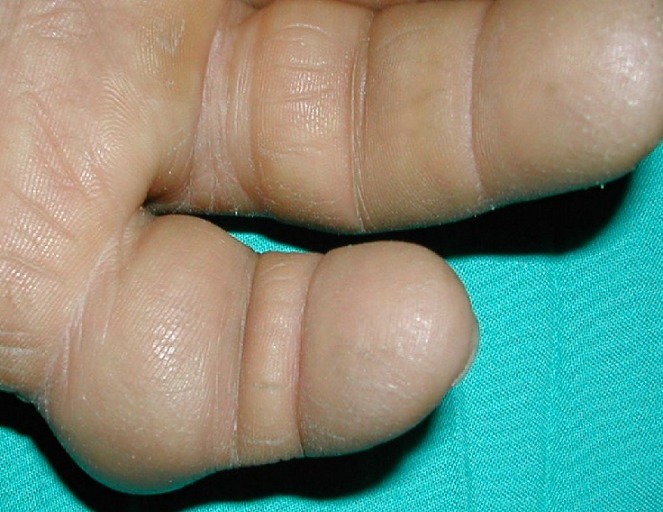
Tuméfaction circonférentielle du 5ème doigt en rapport avec une tumeur à cellules géantes des gaines synoviales de la main

**Figure 2:**
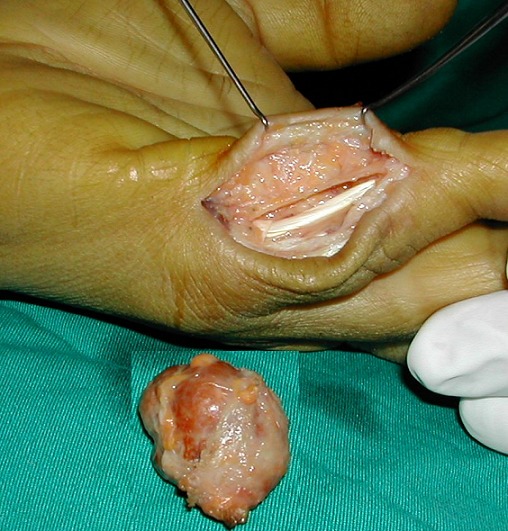
Vue per opératoire: aspect macroscopique de tumeurs à cellules géantes des gaines synoviales de la main

Il s'agissait dans la plupart des cas d'un adulte jeune de sexe féminin avec 31 femmes et 19 hommes âgés en moyenne de 33 ans (9 ans-69 ans). Le coté dominant était touché dans 31 cas soit 62%. La tumeur était peu symptomatique expliquant ainsi la longue latence diagnostic, qui est en moyenne de 18 mois avec des extrêmes de 2 mois et 8 ans. Les motifs de consultation étaient l'apparition constante d'une tuméfaction (100%), la gêne à la mobilisation des articulations inter-phalangiennes (6%) et la douleur digitale (18%). Un patient s'est présenté pour une première récidive. Sur le plan topographique, la tumeur était répartie comme suit : 26% au niveau du pouce, 42% au niveau de l'index, 16% an niveau du médius, 12% au niveau de l'annulaire, et 4% au niveau de l'auriculaire (Figure 1). Elle siégeait en palmaire dans 66% des cas, en dorsale dans 22% des cas et mixte dans 12% des cas (Figure 1). La tumeur était à proximité articulaire dans 20% des cas. La notion de traumatisme était retrouvée dans 13 cas (26%). La radiographie standard centrée sur le doigt malade a été pratiquée de façon systématique chez tous nos patients. Cet examen a inconstamment montré des signes indirects de la tumeur à savoir un épaississement des parties molles dans un tiers des cas, une géode dans 2 cas, et une érosion corticale dans 5 cas. Devant le doute diagnostique avec une tumeur kystique et pour les tumeurs de gros volume, un complément d'exploration par une échographie des parties molles a été réalisé dans 6 cas et par IRM dans 2 cas, confirmant l'aspect d'une tumeur solide, mais sans présomption du diagnostic histologique. Tous les patients ont bénéficié d'une biopsie exérèse devant les arguments cliniques et l'aspect macroscopique per opératoire. Macroscopiquement c'était une masse encapsulée, polylobée et de couleur jaune brunâtre (Figure 2). Des difficultés étaient notées au cours de l'exérèse chirurgicale : quatre tumeurs avaient un prolongement sous le tendon fléchisseur, deux cas avaient un prolongement sous le tendon extenseur, une tumeur était au contact du pédicule digital et une tumeur s'est compliquée d'un envahissement articulaire. Aucun cas d'envahissement osseux ou cutané n'a été noté.

**Figure 3:**
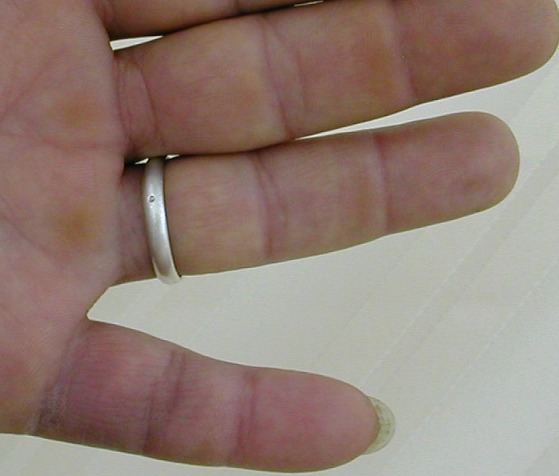
Résultats fonctionnels, récupération complète de la mobilité après la chirurgie de tumeurs à cellules géantes des gaines synoviales de la main

A l'étude histologique, la tumeur est formée de cellules histiocytaires polynuclées dispersées entre des fibres de collagènes avec une hyperplasie très vascularisée de la synoviale. Les macrophages sont pleins de vacuoles lipidiques avec dépôt de quantité variable d'hémosidérine. La tumeur est englobée par une capsule propre avec des prolongements qui pénètrent et cloisonnent la masse en plusieurs nodules de taille variable. Aucun signe de malignité n'a été signalé (atypie nucléaire ou activité mitotique). Après un recul moyen de deux ans (2 mois- 8 ans), nous avons noté une seule récidive (2%) survenue à trois ans postopératoires. Cette récidive a été reprise avec une bonne évolution fonctionnelle. Par ailleurs, nous n'avons pas constaté de complications iatrogènes en particulier nerveuses. La rééducation a été prescrite systématiquement. Le doigt intéressé récupérait sa mobilité complète (Figure 3). Nous avons noté une disparition complète de la douleur et de l'hypoesthésie chez les patients ayant ces deux plaintes avant l'exérèse de la tumeur. Aucun cas d'hypoesthésie ni d'atteinte vasculaire n'a compliqué nos malades. Aucun cas de nécrose cutané n'a été noté. La reprise du travail au même poste était de principe chez les malades travailleurs après une période de convalescence de 21 jours (16 jours à 30 jours).

## Discussion

Nous avons constaté que la plupart des paramètres épidémiologiques de notre série, tels que l´âge moyen, le sexe, le siège de la tumeur, le côté atteint et les signes fonctionnels sont en harmonie avec les autres données de la littérature [6, 7]. La tumeur à cellules géantes des gaines synoviales des mains est une tumeur de l'adulte jeune. Il s'agit d'une pathologie de la 4^ème^ et de la 5^ème^ décade [7, 8]. La quasi-totalité des études ont noté une prédominance féminine [7, 9, 10]. Nous avons retrouvé cette notion dans notre série. Curieusement l'index demeure le doigt le plus touché [7, 8]. Aucune hypothèse n'est avancée dans ce sens. Ces tumeurs se développent généralement en regard des articulations interphalangiennes (IPP-IPD) ou métacarpo-phalangienne (MP), par ordre de fréquence, plutôt en regard de l'IPD, puis la MP et enfin l'IPP. Le diagnostic de la tumeur n'est retenu qu'après un délai souvent longtemps [8, 10]. Comme la plupart des tumeurs des parties molles, l'étiologie des TCGGS de la main reste méconnue. Il s'agit d'une tuméfaction digitale, généralement unique, indolore dans la majorité des cas, souvent palmaire, ferme, bien limitée, de taille variable, à pouvoir évolutif lent et mobile par rapport au plan superficiel. A

la phase clinique, le diagnostic différentiel se pose souvent avec les granulomes à corps étranger de la main, les fibromes des gaines tendineuses, le kyste anévrysmal, le lipome ou une infection [11]. La radiographie standard, faite systématiquement chez tous nos patients, recherche des anomalies caractéristiques à type d'épaississement, érosion corticale ou calcifications. Les érosions osseuses sont à bords nets et mesurent quelques millimètres. Elles sont secondaires soit à l'effet de la pression exercée par la masse tumorale, soit à la résorption secondaire à l'activité ostéoclastique des cellules géantes [12]. Dans notre série, des modifications radiologiques étaient notées dans 22 cas : un épaississement des parties molles dans 15 cas, des géodes de la phalange dans 2 cas et une érosion corticale dans 5 cas. Park et al [13] ont noté 15 lésions osseuses sur 155 cas (11%). De Shepper et al [14] ont rapporté 6 lésions radiologiques concomitantes de TCGGS, avec 4 défects corticaux et deux ostéolyses.

Ces auteurs soulignent l'intérêt de l'IRM pour le diagnostic. L´échographie permet de confirmer la nature tissulaire de la tumeur sans préjuger de son étiologie. Elle décèle la nature kystique d'un kyste synovial qui peut simuler une TCG. Elle permet également de chercher les lésions satellites et d'étudier les rapports de la tumeur avec les structures avoisinantes [15]. Pour Middelton et al [15], la TCGGS se présente à l'échographie comme une masse homogène hypoéchogène liée aux tendons, avec détection de flux sanguin au doppler artériel. L'IRM est le meilleur examen pour une excellente analyse de la gaine tendineuse. Elle précise la taille et le siège exacte de la tumeur ainsi que ses rapports. La tumeur a des limites discrètement bien définies. Elle est bien caractérisée car se développe au contact d'une gaine synoviale. Elle apparait en hyposignal en séquence pondérée en T1 et en hypersignal en séquence pondérée en T2, elle se rehausse de manière diffuse après injection de gadolinium [9]. La mise en évidence d'hémosidérine affirme le diagnostic. En effet, l'hémosidérine reste en hypo-signal [9]. Certains auteurs [16], ont montré l'intérêt de l'étude cytologique du produit d'aspiration de la tumeur à l'aiguille. En effet si cette technique n'offre pas une certitude diagnostique du type histologique, elle permet au moins de s'assurer de l'absence d'atypies cellulaires en rapport avec un sarcome. La tumeur ténosynoviale à cellules géantes reste une lésion de nature inconnue avec un taux de récidive locale relativement élevée jusqu´à 45% dans certaines séries [3]. Plusieurs facteurs de récidive ont été rapportés dans la littérature. Rao et Vigorita [3] ont trouvé qu'une cellularité accrue et une activité mitotique élevée étaient fréquentes dans les lésions récurrentes. Cependant, les auteurs ne trouvent aucune relation claire entre le nombre de mitoses et le taux de récidive. D´autre part, Kitagawa et al. [17] et Al-Qattan [2] ont signalé que ni l'hypercellularité ni l'index mitotique élevé ne pourraient être considérées comme facteurs histologique pronostiques de récidive. Des études récentes en oncologie moléculaire ont suggéré que nm 23 (gène exprimé dans les cellules normales et peut inhiber l´infiltration) pouvait être utilisé comme marqueur pronostique. Les tumeurs à cellules géantes nm 23 négative sont plus agressives et sont associées à un taux de récidive plus élevé [18]. Jones et al. [11] ont trouvé chez les 53 patients parmi 91 avec TCGGS que l'articulation à partir de laquelle la tumeur a pris naissance ou celle la plus proche de la masse tumorale, avait une preuve clinique et/ou radiologique de lésion dégénérative traumatique ou idiopathique.

Les auteurs ont suggéré que l´articulation endommagée pourrait prédisposer à l´accumulation des histiocytes soit dans la synoviale articulaire ou dans les gaines des tendons adjacents. Dans certaines conditions, ces histiocytes pourraient proliférer, subir une métaplasie et produire une tuméfaction avec un aspect histologique de tumeur à cellules géantes de la gaine du tendon. Les cellules géantes, les fibroblastes ou cellules épithélioïdes sont généralement prédominantes et constituent une grande proportion de la masse tumorale. En outre, la synoviale articulaire ainsi que de la gaine du tendon peuvent abriter les tumeurs, localement agressives, mais le plus souvent indolores avec un potentiel de croissance limité [11]. En dépit de cela, la présence d´une lésion dégénérative des articulations adjacentes augmente la difficulté rencontrée pour obtenir une excision chirurgicale complète de la tumeur [17]. Reilly et al. [4] et Grover et al. [18] ont noté que l´érosion osseuse, trouvée sur les radiographies standards, pourrait être un facteur de récidive. Cependant, Kitagawa et al. [17] n´ont pas soutenu cette théorie. Les auteurs ont suggéré que l'envahissement osseux chez les patients atteints de TCGGS localisées était due à une érosion simple, causée par l´effet de la pression exercée par la masse tumorale, et ce n'est pas une véritable invasion. De même, Al-Qattan [2] a constaté que l'érosion osseuse n´a pas d´effet significatif sur la le taux de récidive. Nous n'avons pas également réussi à trouver une corrélation entre l´érosion osseuse et la récurrence. Le seul cas de récidive de notre série ne présente pas d'anomalies radiologiques.

La classification des tumeurs a aidé à analyser les facteurs de récidive. Byers en distingue deux formes : la forme nodulaire (commune à la main) et la forme diffuse (plus fréquente au niveau des articulations) [2, 19]. Al-Qattan a proposé une classification de ces tumeurs basée essentiellement sur la présence ou non de capsule. Cette classification est à visée pronostique (potentiel de récidive). Il s'agit de type I où la tumeur est entourée par une capsule et le type II correspondant à une tumeur non encapsulée plus pourvoyeuse de récidive [2]. Al-Qattan a étudié 43 cas de TCGGS avec un recul de quatre ans (deux-six ans). Sur 30 tumeurs encapsulées, aucune récidive n'a été notée alors que sur 13 tumeurs non encapsulées il y'a eu cinq récidives. Le traitement chirurgical des TCG a un double objectif : diagnostique et thérapeutique. En effet, il permet de confirmer le diagnostic anatomopathologique de la lésion d'une part et la rémission carcinologique d'une autre. Le traitement est toujours chirurgical qui consiste à une exérèse de la tumeur en totalité. Cette exérèse doit être méticuleuse et complète afin de prévenir la récidive tumorale. Selon Suresh et al, Une exérèse incomplète de la tumeur, due à une technique chirurgicale insuffisante, est la cause la plus importante de récidive [20]. Une exposition chirurgicale adéquate, une dissection méticuleuse et l'utilisation d'un microscope lors de l'exérèse sont nécessaires pour réduire le taux de récidive [20]. Ce taux est diminué d'une manière remarquable si les patients reçoivent une radiothérapie adjuvante [5].

Dans notre série, la récidive est très vraisemblablement due à une exérèse incomplète. Finalement, on peut dire que l'appréciation rigoureuse du risque relatif de récidive de ce type de tumeur permettra de planifier une chirurgie appropriée et d'informer les patients concernant le risque de récidive. La radiothérapie a été proposée suite à l'exérèse difficile et parfois incomplète des tumeurs ayant des prolongements en intra-articulaire, et même en intra-osseux ou autour des pédicules collatéraux ou en présence d'une activité mitotique élevée à l'examen histologique pour prévenir la récidive [5, 18]. Kotwal et al ont rapporté seulement 4% de récidive après radiothérapie adjuvante [5]. Cependant, pour Ozalp et al., L´excision est suggérée, même après plusieurs récidives, et la radiothérapie n'a pas de place même pour les tumeurs récurrentes [6].

## Conclusion

Les TCGGS sont des tumeurs bénignes à malignité strictement locale avec une tendance agressive. Le diagnostic bien qu'il soit tardif, doit être évoqué devant toute tuméfaction digitale, palmaire et/ou dorsale, indolore, évoluant depuis longtemps. Le pronostic évolutif est dominé par le risque de récidive après exérèse chirurgicale. Leur prise en charge fait appel à la chirurgie qui reste difficile et qui doit être bien planifiée et correctement exécutée pour éviter les récidives.

### Etat des connaissances actuelle sur le sujet

- 2^ème^ tumeur la plus fréquente des tumeurs rencontrées au niveau de la main après les kystes synoviaux;
- Evolution non douloureuse ce qui explique la tumeur de taille importante;
- Potentiel de récidive important pouvant aller jusqu'à 45%.

### Contribution de notre étude à la connaissance

- Le diagnostic de tumeur à cellules géantes des gaines synoviales de la main doit être évoqué devant toute tuméfaction digitale;
- Le traitement est exclusivement chirurgical;
- Une exérèse incomplète de la tumeur due à une technique chirurgicale insuffisante, est la cause la plus importante de récidive.
